# Validation of a newly automated web-based 24-hour dietary recall using fully controlled feeding studies

**DOI:** 10.1186/s40795-017-0153-3

**Published:** 2017-04-05

**Authors:** Jacynthe Lafrenière, Benoît Lamarche, Catherine Laramée, Julie Robitaille, Simone Lemieux

**Affiliations:** 1 School of Nutrition, Laval University, Québec, Qc Canada; 2 Institute of Nutrition and Functional Foods, Laval University, Québec, Qc Canada

**Keywords:** Food assessment, Dietary recall, Portion size estimation, Validation, Feeding studies

## Abstract

**Background:**

Assessment of food intake is a cornerstone of nutritional research. However, the use of minimally validated dietary assessment methods is common and can generate misleading results. Thus, there is a need for valid, precise and cost-effective dietary assessment tools to be used in large cohort studies.

The objective is to validate a newly developed automated self-administered web-based 24-h dietary recall (R24W), within a population of adults taking part in fully controlled feeding studies.

**Methods:**

Sixty two adults completed the R24W twice while being fed by our research team. Actual intakes were precisely known, thereby allowing the analysis of the proportion of adequately self-reported items. Association between offered and reported portion sizes was assessed with correlation coefficients and agreement with the kappa score while systematics biases were illustrated with Bland-Altman Plot.

**Results:**

Participants received an average of 16 food items per testing day. They reported 89.3% of the items they received. The more frequently omitted food categories were vegetables included in recipes (40.0%) as well as side vegetables (20.0%) and represented less than 5% of the actual daily energy intake. Offered and self-reported portion sizes were significantly correlated (*r* = 0.80 *P* < 0.001) and demonstrated a strong agreement as assessed by the kappa score of 0.62. Reported portion sizes for individual food items were on average 3.2 g over the offered portion sizes. Portions of 100 g and above were on average underestimated by 2.4% (*r* = 0.68 *P* < 0.01; kappa score = 0.50) while small portions (less than 100 g) were overestimated by 17.1% (*r* = 0.46 *P* < 0.01; kappa score = 0.43). A nonsignificant underestimation (−13.9 kcal ± 646.3 kcal; *P* = 0.83) of energy intake was noted.

**Conclusion:**

R24W performed well as participants were able to report the great majority of items they ate and selected portion size strongly related to the one they received. This suggests that food items are easily to find within the R24W and images of portion sizes used in this dietary assessment tool are adequate and can provide valid food intake evaluation.

**Electronic supplementary material:**

The online version of this article (doi:10.1186/s40795-017-0153-3) contains supplementary material, which is available to authorized users.

## Background

High quality nutritional research hinges on valid assessment of food intake. However, it remains a real challenge to adequately measure food intake. Because of wide within subject variation, self-reported tools have some degree of random errors [1] often associated with incoherent research results [2]. Recent researches demonstrated that those errors may be significantly reduced by improving data collection techniques and by selecting tools adapted to the studied population. Furthermore, it is essential to validate a new dietary assessment tool before its first use [3].

Self-reported food assessment tools are often associated with high rates of misreporting leading to underestimation of energy and nutrient intakes compared to objective measurements [4]. Misreporting can be explained in part by undereating and in part by under-recording. Indeed, subjects tend to reduce or change their food intake when they have to report it. This has been referred to as the reactivity bias [5]. In addition, studies have shown that subjects frequently fail to remember all the items they ate and they have trouble estimating the exact portion sizes consumed [6–8]. These errors could be attributed to memory or social desirability bias [4, 9].

Automated self-administered 24-h recalls received attention lately and are increasingly used because there are convenient and cost-effective [10]. For cohort studies, automated 24-h dietary recall is becoming the tool of choice instead of food frequency questionnaire because of its superior precision and accuracy [11, 12]. Furthermore, it presents characteristics that can help reducing the above-mentioned biases. First, as participants generally filled their recalls on unannounced days, this limits the reactivity bias. Second, those recalls are completed by the respondent, outside of a laboratory setting, thus in a neutral environment [13]. This reduces the social desirability bias as compared with a face to face administration of recalls [14, 15]. Third, the inclusion of memory cues in the recall can attenuate memory bias which can therefore contribute to reduce underreporting. For example, with an approach like the USDA automated multiple pass method, items are reviewed to make sure that nothing has been forgotten, context of the meal is accentuated because it helps to remember details about the food consumed and many questions are asked about frequently forgotten food items [16]. In addition, images of portion sizes can improve estimation accuracy by up to 60% [17, 18]. Finally, presentation of simultaneous different portion size options can reduce error rates compared to presentation of only one option [7].

Some studies have shown that, compared with the traditional interview, web-based 24-h recall generates equivalent results with a reduced precision for some nutrients [6, 19, 20]. However, the preference for the web-based version was highlighted by many researchers. Indeed, Thompson et al. [19], showed that using a web-based 24-h recall reduced attrition rate compared to interview administered 24-h recall. Furthermore, with the web-based approach, the completion time is reduced and the coding step is automated thereby saving a significant amount of time [21]. The main concern remaining is the accessibility as internet connexion is not yet equivalently spread causing an underrepresentation of older adults with lower incomes (in 2012, 28% of them had internet access compared with 95% of younger adults with higher incomes [22]).

It has to be emphasized that when a new dietary assessment tool is developed, a rigorous validation process needs to be performed before it can be used in cohort studies. One strategy that can be used when validating dietary assessment tools is to compare self-reported food intake to the actual food intake consumed in the context of fully controlled feeding studies. In these projects intended to evaluate the effects of specific nutritional manipulation, participants receive all their meals for the duration of the study. The specific composition and weight of each food item consumed is therefore known and can be compared to the recall filled by the participants afterwards.

The R24W is a newly developed web-based, self-administered and fully automated 24-h recall [23]. It is the first French-language web-based automated 24-h recall developed to assess dietary intake in the French-Canadian population. The aim of this study was to validate the R24W in a context of fully controlled feeding studies. More precisely, we wanted to evaluate adequate reporting of food items and portion size evaluation. We hypothesize that the majority of offered food items are adequately reported using the R24W. We also hypothesize that there is a portion size estimation error of less than 10%. Furthermore, as other authors reported that adults tend to underestimate large portion sizes compared with smaller ones [24, 25] we decided to test specifically error rate in small and larger portions and we hypothesize that, because of the large distribution of portion sizes illustrated in the R24W, the difference between them is not significant.

## Methods

### Participants

This analysis was conducted on 33 men and 29 women already enrolled in three fully controlled feeding studies currently conducted in our research institute. To be included in these studies, they had to be non-smoking men or women aged between 18 and 75 years old with stable weight. Women should not be pregnant or lactating. Each participant had to be free from cardiovascular or endocrine diseases and should not have a food allergy or aversion to any food item offered in the feeding protocol. These studies received the approval of the Laval University Ethics Committee and participants provided written informed consent prior to taking part in the study. The analysis performed in this article was directly presented in one of the three consent forms (the latest study) and was proposed to the participants already included in the two other studies in an addendum to their initial consent form. Therefore, all participants gave their informed consent before completing their first 24-h recall knowing that it was part of a validation process.

These clinical trials were registered at http://www.clinicaltrials.gov as NCT02763930, NCT02106208 & NCT02029833. Participants followed the initial research protocol but in addition to this, we asked them to fill the R24W twice.

### The automated web-based automated 24-h recall (R24W)

Details on how the R24W was developed have been published elsewhere [23]. Briefly, R24W was developed in French language and was inspired by the AMPM of the USDA [16]. However, as opposed to AMPM, R24W is using a meal-based approach in the first step. An unlimited number of meals or snacks per 24-h period can be added by the respondent. R24W allows automatic calculation of different diet quality scores in addition to energy and nutrient intakes. The application was programmed in such a way that the days for which the recall has to be completed are randomly generated with the possibility of using specific criteria (e.g., proportion between weekdays and weekend days). For this validation study, the same two days in each study were used for recalls. The application also allows the automatic sending of email messages to participants prompting them to complete their 24-h food recall. The database includes 2865 items linked to the Canadian Nutrient File (2010 version) or the USDA Nutrients Database for the few items that were not available the Canadian Nutrient File. Questions about the context of the meals are asked to help respondents to recall all the items consumed. There is also systematic questions about frequently forgotten food items. Portion sizes are represented by up to eight food pictures representing predetermined portion sizes in a fixed neutral set-up. Portion sizes are expressed in units and/or volume under each picture. Respondents also have the option to select a multiplicative or a fraction of each portion shown. The format has been designed to be intuitive. In a pre-test in a cohort of 29 adults with different levels of computer skills, the R24W was found to be easy to understand and to complete [23].

### Research context

Studies in which participants were initially involved had different feeding protocols. Most of these studies aimed at assessing the metabolic effects of some diets for which nutrient composition was manipulated. Menus were formulated using typical French Canadian food items. Meals were prepared and provided by the research team according to a 7-day cyclic menu for 4 to 8 weeks followed by 4 to 8 weeks of wash-out where subjects returned to their normal diet. The portion sizes of the different food items offered were individualized to ensure that all participants maintained a stable body weight while the exact diet composition was kept the same for each experimental condition in each study. Participants were not aware of the precise amount of food they received. They were instructed to eat all the food items provided every day and nothing else. Participants’ body weight was measured throughout each project to achieve isoenergetic conditions and energy intake was increased or decreased by 250 kcal/day if a subject lost or gained greater than 1 kg and maintained that body weight for at least 3 days. In order to standardize the testing between studies, the R24W was completed in the first two weeks of one of the feeding phases for each project. Menus were composed of three meals and a snack per day. Except for a few exceptions, lunch (40% of daily calories) was consumed in the lab facility while dinner, breakfast and snack were packed in a cooler and consumed outside the clinical facility. Items were all labelled and participants received a checklist to remind them to eat the entire menu in order to enhance compliance (see Additional file 1). On this checklist, the general name of the meals and side dishes were given but the list of ingredients for mixed dishes was not included. This gave us the opportunity to assess how well participants managed to find the food items they consumed among the list of food available in the R24W with a reduced memory bias. Since no cues were provided on the check list about portion size of food items consumed, a memory bias could however influence how subjects were choosing the portion sizes when filling the R24W.

### Validation strategy

In each study included in this protocol, the nutrient composition of the meals were manipulated without the knowledge of the participants. Therefore, it would not be the most appropriate study design to evaluate the accuracy of reported nutrient intakes. Instead, we decided to use a validation strategy in which we compared food items reported to food items actually offered. This was done by classifying reported food items as “matches”, “omissions” and “inclusions” as previously suggested [6]. A “perfect match” corresponded to a situation where subjects selected the exact appellation of the item they received in the R24W (e.g., boiled potatoes for boiled potatoes). A “close match” described the selection of an item with related characteristics (e.g., mashed potatoes instead of boiled potatoes). A “far match” was used to classify an item in the same food categories but with different characteristics and nutritional composition (e.g., fried potatoes instead of boiled potatoes). An “omission” was used to define a food item that was provided but not reported. Finally, an “intrusion” corresponded to a food item that was reported but not provided. For all items classified as matches (either perfect, close or far), offered and reported portion sizes were compared. Finally, only for indicative purposes, energy and macronutrient intakes as reported by participants who filled the R24W were compared with actual energy and macronutrient intakes as provided by the menu offered.

### Statistical analyses

Proportions of matches and omissions were calculated and the average number of inclusions was reported. Omissions were then analyzed in-depth to determine which categories of food items were more frequently omitted by participants. The impact of these omissions on energy intake assessment was then evaluated. To do so, we first classified the omitted items by categories and then calculated the mean contribution to the daily energy intake of all the mentioned items in this category. More precisely, the energy content of each item was determined and then a weighted average was calculated to represent the contribution of the category to the daily energy intake. As all menus were standardized, food items from a given food category had the same relative contribution to the total energy intake for all participants.

The difference between each reported and offered portion size was assessed (absolute number) and, in the present study, we refer to this difference as the bias which provides an indication of the systematic underestimation (in case of a negative bias) or overestimation (in case of a positive bias) in portion sizes. The difference between reported and offered portion size was also characterized as the estimation error that is the ratio between the bias and the offered portion size (in percentage). A Student *T*-Test was used to compare mean portion size reported to mean portion size offered. Analysis were conducted on all data and also separately on the smaller portions (characterized as less than 100 g) and on the larger portions (characterized as 100 g and above). A Student *T*-test was performed to determine if a larger estimation error occurs in larger portion sizes compared to smaller portion sizes. To assess accuracy in portion size estimation, we used correlation coefficients, weighted kappa scores for classification in quartiles and the Bland-Altman plots. The Kappa score describes the agreement between two measures as poor (<0.00), slight (0.00–0.20), fair (0.21–0.40), moderate (0.41–0.60), substantial (0.61–0.80) and almost perfect (0.81–1.00) [26]. In order to investigate if individual characteristics could influence portion size estimation, a stepwise linear regression model was tested with age, sex and BMI as predictive factors for estimation error.

In the controlled feeding trials, lunch was served on a plate and consumed at the research institute while dinner was provided in individual plastic containers to be taken home. The extent to which meal presentation affected the accuracy of portion size estimation with the R24W was also assessed using T-tests comparing estimation bias between meals. Finally, energy and macronutrient intakes reported were compared to values corresponding to the offered food items using a Student *T*-test. Correlation analyses were also performed between reported and offered energy and nutrient intakes. Statistical analyses were conducted with the software SAS version 9.4 (SAS Institute).

## Results

Characteristics of the participants are shown in Table 1. Data from 62 adults aged 21 to 71 years with body mass index from 21 to 52 kg/m^2^ are included in the analyses.

**Table 1:** Characteristics of the participants (*n* = 62)

	*N* (%)
Sex	
Men	33 (53.2%)
Women	29 (46.8%)
Mean BMI	
Normal weight (18.5–24.9 kg/m^2^)	7 (11.3%)
Overweight (25.0–29.9 kg/m^2^)	25 (40.3%)
Obese (30 kg/m^2^ and above)	30 (48.4%)
Mean age (y)	
< 25	5 (8.1%)
25–50	30 (48.4%)
> 50	27 (43.5%)

Participants received on average 16 different food items per day. The proportion of matches and omissions as well as the number of intrusions in participants’ responses to the R24W are presented in Table 2. A total of 89.3% of the offered food items were reported with the R24W and 76.8% were reported using the exact descriptor in the database. Descriptions of the main omissions are presented in Table 3. Omissions were classified according to different food categories and the weighted average contribution of the category to the offered daily energy intake was calculated. Finally, Table 3 indicates whether the omitted food items were specifically named in the checklist that was provided to participants.

**Table 2:** Proportion of matches (exact, close and far) and omissions related to the amount of food items offered and number of intrusions for all subjects^a^ (*n* = 62)

	Proportion or number of items
Number of items offered/day (*n*)	16.1 ± 3.1
Exact matches (%)	76.8 ± 15.3
Close matches (%)	8.2 ± 8.7
Far matches (%)	4.3 ± 5.2
All matches combined (%)	89.3 ± 11.1
Omissions (%)	10.7 ± 11.1
Intrusions (*n*)	0.2 ± 0.7

^a^Perfect match: a situation where subjects selected the exact appellation of the item they received in the R24W. Close match: an item with related characteristics. Far match: an item in the same food category but with different characteristics and nutritional composition. Omission: a food item that was provided but not reported. Intrusion: a food item that was reported but not provided

**Table 3:** Counts of the items most frequently omitted by participants in relation to offered items

Food items	Number of subjects who received the item	Items included in the checklist	Number of omissions	Mean contribution to the daily energy intake^b^
Vegetables in a salad or a mix dish			72	0.7%
Peppers	45	No	24	
Celery	32	No	17	
Cucumbers	13	No	11	
Corn	26	Yes/No^a^	10	
Onions	13	No	7	
Tomatoes	13	Yes	3	
Side vegetables			36	3.1%
Sweet potatoes	32	No	21	
Potatoes	32	Yes	4	
Coleslaw	32	Yes	3	
Tomatoes	13	Yes	2	
Cucumbers	13	Yes	2	
Broccoli	18	Yes	2	
Cauliflower	18	Yes	2	
Snacks/drinks			30	6.2%
Cheddar cheese	19	Yes	7	
Sweet bread/muffin	73	Yes	6	
Raspberries	18	Yes	6	
Milk	18	Yes	3	
Milk shake	26	Yes	3	
Yogurt	31	Yes	3	
Blueberries	18	Yes	2	
Sauces			26	1.8%
Vinaigrette	13	Yes	7	
Salsa	13	Yes	7	
BBQ sauce	32	Yes	6	
Mayonnaise	13	No	6	
Ingredients in a salad			16	3.7%
Feta cheese	13	No	10	
Cranberries	13	Yes	3	
Chicken	13	Yes	3	

^a^Corn was offered in two different menus, one where it was written as an ingredient on the checklist (3 omissions/13 presentations) and one where it was not included on the checklist (7 omissions/13 presentations)

^b^The energy content of each item was determined and then a weighted average was calculated to represent the energetic contribution of the category

Table 4 presents examples of small and large portions offered at breakfast and at lunch/dinner meals. Most of the main dishes were offered in portions larger than 100 g while side vegetables, fruits, sauces and spreads were mostly in portions smaller than 100 g.

**Table 4:** Examples of small and large portions of food items offered^a^

	Small portions (g)		Large portions (g)	
Breakfast	Bread/bagel	27–88	Orange	100–320
	Peanut butter	16–50	Milk	125–400
	Ham	12–40	Milkshake	130–416
	Cereals	30–96	Orange juice	160–512
	Cream cheese	8–26	Apple sauce	125–400
	Raspberries	25–80		
	Blueberries	27–88		
Lunch/Dinner	Cranberries	8–25	Vegetable juice	125–400
	Cucumbers	15–48	Rice with shrimps	320–570
	Tomatoes	25–80	Potatoes	172–307
	Vinaigrette/mayo	10–32	Fajitas with beans	200–355
	Cheese	20–64	Meat loaf	165–528
	Broccoli	22–72	Pesto pasta	207–368
	Carrots	22–72	Chili con carne	330–840
	Salsa	14–45	Mexican turkey	108–270
			Parsley salad	165–528
			Ham quiche	102–256
			Roasted peppers	97–272
			Carrot soup	165–528
			Rice	129–292
			Couscous	137–309

^a^A range is presented as portions were individualized according to participants’ energy needs varying from 1750 to 4500 kcal per day

Table 5 describes the agreement between reported and offered portion sizes (kappa score, correlation coefficient and estimation bias). Analyses were first conducted with all portions irrespective of their size and then with small and large portion sizes separately. When analysing all portions irrespective of their size, we found a small nonsignificant (*P* = 0.12) systematic bias of 3.2 g (9.3% ±66.0%) meaning that R24W tends to slightly overestimate portion sizes. The correlation coefficient of 0.80 and the weighted kappa score of 0.62 suggest a substantial agreement between reported and offered portion sizes. Offered portions of less than 100 g (kappa score of 0.43) as well as portions larger than 100 g (kappa score of 0.50) showed a moderate agreement with their corresponding reported portion sizes. The estimation error was significantly larger for small (17.1% ±78.5%) than for large portions (−2.4% ±55.8%; *P* < 0.01).

**Table 5:** Agreement between reported and offered portion sizes as determined by the Kappa scores for portion size classification in quartiles, correlation coefficients and estimation bias for all, small and large portions

	Kappa score	Correlation coefficient (*P*)	Estimation bias (estimation error)^a^
All portions *N* = 1373	0.62	0.80*	3.2 g (9.2%)
Small portions (<100 g) *N* = 640	0.43	0.46*	7.6 g* (17.1%)
Large portions (≥100 g) *N* = 733	0.50	0.68*	−0.6 g (−2.4%)

^a^Estimation bias: the average of the difference between reported and offered portion size. Estimation error: the mean ratio between the bias and the offered portion size

*Significant at *P* < 0.05 (Difference between reported and offered portion was calculated to obtain the estimation bias)

**Fig. 1:**
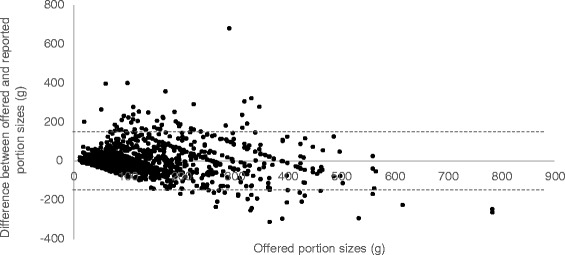
Bland Altman Plot of the comparison between offered and reported portion sizes for all portions. Bias = 3.2 g Limits of agreements (*dotted line*): −148.3 to 154.7 g R: −0.15 *P*: <0.001

**Fig. 2:**
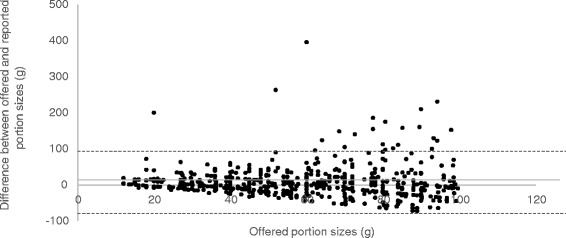
Bland Altman Plot of the comparison between offered portions of less than 100 g and reported portion sizes Bias = 7.6 g Limits of agreements (*dotted line*): −76.9 to 92.1 g R: −0.02 *P* = 0.54

**Fig. 3:**
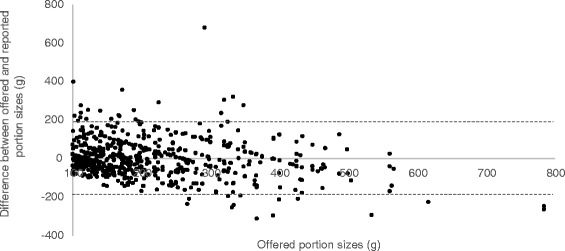
Bland Altman Plot of the comparison between offered and reported portion sizes of 100 g and larger. Bias = −0.6 Limits of agreements (*dotted line*): −192.1 to 190.8 g R: −0.18 *P* < 0.001

Figures 1, 2 and 3 illustrate the Bland-Alman plots for differences between reported and offered portion sizes for all food items, and also for small portions and large portions separately. Because the exact offered portions were known, these were used as the independent variables. The plots demonstrated acceptable agreement. The linear regression model showed that neither age, sex nor BMI were significantly predicting the estimation error in this sample. Results showed that there was no significant difference in the estimation error between meals offered for lunch (2.9 g ± 134.8 g) and meals offered for dinner (−11.0 g ± 93.7 g), *P* = 0.29 (not shown).

Finally, comparisons between reported and offered energy and macronutrients are presented in Table 6. Significant correlations were observed (*r* = 0.38–0.64 *P* < 0.01) and reported energy and protein intakes were not statistically different from the offered amounts. However, reported carbohydrate intakes were significantly lower while reported fat intakes were significantly higher than offered values.

**Table 6:** Comparison between reported and offered intakes for energy and macronutrients (*n* = 62)

	Reported intake	Offered intake	Difference between reported and offered intakes	Correlation coefficient between reported and offered intakes
Energy (kcal)	2762.4 ± 781.1	2776.4 ± 603.8	−13.9 ± 646.3	0.59*
Proteins (g)	110.9 ± 39.2	110.0 ± 25.2	2.1 ± 26.3	0.60*
Carbohydrates (g)	340.9 ± 101.6	366.9 ± 75.9	−26.1 ± 79.0*	0.64*
Fat (g)	111.0 ± 32.0	102.8 ± 26.6	9.1 ± 38.0*	0.38*

*Significant at *P* < 0.05

## Discussion

The objective of this study was to validate in terms of food items reporting and portion size evaluation a new web-based 24-h recall, the R24W, using the context of fully controlled feeding studies. We observed that the majority of the offered food items – close to 90%, were reported by the participants and that the mean difference between offered and reported portion sizes was less than 10% (i.e., 9.3%), as we hypothesized. This slight difference resulted in a non-significant 13.9 kcal underestimation of energy intake. However, contrary to our initial assumptions, we observed that portions smaller than 100 g were estimated at a greater error rate than those of 100 g and above.

Nutritional assessment errors can be attributed to participants’ recall bias (reactivity, memory or social desirability bias) or to inherent characteristics of the tool (e.g., inadequate strategy for data collection or visual support). While it is difficult to distinguish them in a validation study conducted in the context of real life, fully controlled studies allow to minimize some participants’ related bias. However, few studies have used controlled feeding studies to validate automated self-administered 24-h dietary recalls. Indeed, tools are generally compared to another self-reported tool or with biomarkers that provide precise information about only one nutrient at a time [13]. Kirkpatrick et al. conducted a similar study intended to validate the ASA24 [6], another web-based dietary recall, in a context where subjects were invited to eat in a research cafeteria setting and asked to report their intakes during a follow-up visit, the day after. They observed that the proportion of matches between food consumed in the lab facility and food reported was 80%, a value that is slightly lower than what we observed in our study (i.e., 89%). However, memory was more of a confounding factor in that study because subjects were exposed to the experimental meals for the first time and did not have any cues to help them remember their past intakes. This was not the case in our study since subjects might have received the same meals in the previous phase of their studies and because they had access to a checklist to help them remember to consume all food items.

Omitted items identified in the present study were different from those reported in previous studies. Underreporting of fat and carbohydrates with food records and 24-h recalls have been repeatedly demonstrated [5, 27]. This is supported by studies showing high-fat foods such as cakes, pastries, cookies and savoury snacks are more often underreported than other food groups [28, 29]. However, it is important to note that in the present study, the variety of snacks and desserts was limited as participants were imposed a specific menu. Nevertheless, they received cakes and potato chips and those items were not among the most frequently omitted food items. Hebert et al. [9] suggested that underreporting is associated with social desirability, which may explain that typical unhealthy foods tend to be underreported or omitted. In our study, this bias was limited because participants did not have to take responsibility for their food choices, which were predetermined as part of the experimental procedures. As suggested by others, the web interface may also contribute to reducing the social desirability bias compared with a human interviewer [14, 15] but our study was not designed to specifically address this issue.

The most frequently omitted items by our participants were vegetables included in recipes (72 omissions) as well as side vegetables (36 omissions). This is in accordance with the observations of Kirkpatrick et al. [6]. Some of these items were not extensively described in the checklist that was provided to the participant (87 items not described in the checklist/108 omissions in vegetables included in meals and side vegetables), so a memory bias could in part explain these omissions. We can also suggest that for these omitted food items the checklist was misleading in a way, suggesting to the participant that some items were more important than others. For example the “Mexican tortillas” included peppers and onions even if it was not mentioned on the checklist (see Additional file 1). We calculated that the energy contribution of vegetables included in recipes and side vegetables was however minimal (i.e., less than 5% of the daily energy intake).

When analysing all food items, irrespective of the portion size, we noted a mean estimation bias of 3.2 g, which is close to the −3.7 g differences observed by Kirkpatrick et al. [6]. Williamson et al. [30] suggested that digital photos helped to accurately estimate portion sizes of food items. In the present study, participants tended to overestimate small portion sizes and to underestimate larger portions. This observation has also been highlighted in a previous study by Nelson et al. [31]. However, our estimation error in large portion size was only −2.4% suggesting that we selected enough pictures of larger portion sizes [24].

Our results suggest that images used to illustrate the portion sizes in the R24W contribute to influencing adequately estimation of real intakes. It is of importance to remember that participants did not receive information about the size of the portion they ate. Moreover, they received their lunch meal in a plate similar to the one that appears on R24W portion size images while breakfast and dinner were served in plastic containers where items were often mixed, which can increase the difficulty in assessing the portion sizes of each ingredient. However, our results showed that there was no difference in portion size estimation that was noted between presentation formats. As meals are not always consumed in a plate in real life settings, it is of interest to assess portion sizes estimation in different contexts.

The observed overestimation of small portions deserves attention. In fact, the estimation error was close to 20% and the correlation coefficient between offered and reported portion sizes was below 0.50 [32]. Although it was not a specific objective of the study due to the blind manipulation of some food items, the analysis comparing reported and offered energy seems to bring confidence that estimation errors are counterbalanced by omissions, or are of low importance in the estimation of energy intakes. Indeed, the items most frequently overestimated are the same as those most frequently omitted (vegetables, sauces and spread) and the overall energy intake as obtained from the R24W filled by participants is not significantly different from the energy content of the diet offered. However, some differences were found between reported and offered amounts of carbohydrate and fat. These differences can be explained, at least partially by the fact that participants were unaware of the dietary manipulation of the meals they received. For example, in two out of three studies, they received a milkshake supplemented in fat to assess the metabolic effect of different types of oils. Meanwhile, the proportion of fat in other recipes, like muffins was reduced for balancing the diet. These manipulations could explain some of the differences observed between reported and offered amounts of carbohydrate and fat.

Another limitation to consider is the burden associated with self-reporting food intake for many days. In the context of this study, participants were asked to fill the R24W twice on top of the initial research requirements. They were highly motivated and did not seem to significantly underreport. However, it will be a concern with wider and more diversified cohorts. Indeed, considering that at least 3 non-consecutive days of 24-h recall are needed to represent typical intakes [33], the time commitment for participants could impact their motivation. Nevertheless, the web-based format of the R24W seems to be an important asset. Based on a pre-test conducted during its development, 59% of responders completed the recall in less than 30 min [23]. In comparison, the food frequency questionnaire used recently in our large cohort studies takes on average 45 min to complete [34]. Moreover, as the coding is automated, the web-based approaches lead to a considerable time saving for researchers.

## Conclusions

To conclude, when controlling in part for some of the personal bias (memory, reactivity and social desirability), participants reported most of the items they ate with a good level of accuracy in portion sizes reported. This data also provides preliminary evidence supporting the validity of R24W to assess food intakes. Analyses in larger cohorts of free living individuals including biomarker analysis would be completed soon. This will allow to validate dietary assessment with a larger variety of consumed food items. In the near future, the R24W could be used to assess the food intake in large-scale research projects and in nutrition practice.

## Additional file

Additional file 1: Word document (.docx) Annexe 1: Checklist offered to the participant with their meals for each project. (DOCX 13 kb)

## Abbreviations

BMI: Body mass index
R24W: Newly developed automated self-administered web-based 24-h dietary recall
