# Supplementary material for: Validation of a newly automated web-based 24-hour dietary recall using fully controlled feeding studies
Source: BMC Nutr. 2017 Apr 5;3:34. doi: 10.1186/s40795-017-0153-3 (PMC7050885; doi:10.1186/s40795-017-0153-3)
Supplement: Supplementary file 1 — Word document (.docx) Annexe 1: Checklist offered to the participant with their meals for each project. (DOCX 13 kb) [file 40795_2017_153_MOESM1_ESM.docx]

Annexe 1: Checklist offered to the participant with their meals for each project.

| **Project** | **Day tested** | **Meals** | **Food items** |
| --- | --- | --- | --- |
| **1** | **Wednesday** | **Breakfast** | Multigrain bread  Creton  Orange  Milk |
|  |  | **Snack** | Raisin cookie |
|  |  | **Lunch** | Lime and beans fajitas  Tomatoes and cucumbers  Blueberry square  Milk |
|  |  | **Dinner** | Meatloaf with tomato sauce  Pesto fusilli  Chocolate cake  Milk |
|  | **Thursday** | **Breakfast** | Muslix cereals  Milk  Roasted peanuts  Red grapes |
|  |  | **Snack** | Banana bread |
|  |  | **Lunch** | Shrimp rice  Vegetable juice  Graham cake / raspberries  Milk |
|  |  | **Dinner** | Chicken, BBQ sauce  Potatoes  Coleslaw  Chocolate pudding  Milk |
| **2** | **Tuesday** | **Breakfast** | Granola  Raspberry and blueberry yogurt |
|  |  | **Snack** | Oat muffin |
|  |  | **Lunch** | Carrot soup  Chili con carne  Basmati rice  Cranberry bread |
|  |  | **Dinner** | Curry chicken  Couscous  Broccoli and cauliflower  Chocolate cake |
|  | **Sunday** | **Breakfast** | Multigrain bread  Cretons  Apple sauce  Orange juice |
|  |  | **Snack** | Molasse muffins |
|  |  | **Lunch** | Ham quiche  Taboulé salad  Vanilla pudding |
|  |  | **Dinner** | Lemon chicken  Garlic pasta  Roasted peppers  Carrot cake |
| **3** | **Tuesday** | **Breakfast** | Apple muffins  Vanilla Greek yogurt  Strawberry-peach milkshake |
|  |  | **Snack 1** | Red grapes |
|  |  | **Lunch** | Chicken and cranberry spinach salad  Balsamic vinaigrette  Crackers |
|  |  | **Dinner** | Chili con carne  Cheese  Plain chips |
|  |  | **Snack 2** | Date cookie  Berry milkshake |
|  | **Sunday** | **Breakfast** | Oat bran bagel  Cream cheese  Strawberry coulis  Banana milkshake |
|  |  | **Snack 1** | Yogurt |
|  |  | **Lunch** | Turkey sandwich  Humus  Raw vegetables  Orange |
|  |  | **Dinner** | Mexican tortillas  Tomato and corn salad  Salsa vinaigrette |
|  |  | **Snack 2** | Banana muffins  Strawberry-blueberry milkshake |
